# Investigation on Microparticle Transport and Deposition Mechanics in Rhythmically Expanding Alveolar Chip

**DOI:** 10.3390/mi12020184

**Published:** 2021-02-12

**Authors:** Jun Dong, Yan Qiu, Huimin Lv, Yue Yang, Yonggang Zhu

**Affiliations:** 1 School of Science, Harbin Institute of Technology, Shenzhen 518055, China; d.j.dong@foxmail.com (J.D.); qiuyan@zju.edu.cn (Y.Q.); huimin2019@foxmail.com (H.L.); 2 School of Mechanical Engineering and Automation, Harbin Institute of Technology, Shenzhen 518055, China

**Keywords:** microfluidics, alveolar chip, particle tracking, dynamic similarity, high-speed camera

## Abstract

The transport and deposition of micro/nanoparticles in the lungs under respiration has an important impact on human health. Here, we presented a real-scale alveolar chip with movable alveolar walls based on the microfluidics to experimentally study particle transport in human lung alveoli under rhythmical respiratory. A new method of mixing particles in aqueous solution, instead of air, was proposed for visualization of particle transport in the alveoli. Our novel design can track the particle trajectories under different force conditions for multiple periods. The method proposed in this study gives us better resolution and clearer images without losing any details when mapping the particle velocities. More detailed particle trajectories under multiple forces with different directions in an alveolus are presented. The effects of flow patterns, drag force, gravity and gravity directions are evaluated. By tracing the particle trajectories in the alveoli, we find that the drag force contributes to the reversible motion of particles. However, compared to drag force, the gravity is the decisive factor for particle deposition in the alveoli.

## 1. Introduction

The deposition of inhalable particles in human alveoli is closely related to the development of some diseases like chronic obstructive pulmonary disease (COPD), asthma, respiratory infections and lung cancer [1]. The inhalation therapy through respiratory system is currently receiving more attention [2,3,4]. Therefore, it is necessary to study the transport and deposition of particulate matter in human alveolar region, which holds great significance for both disease diagnosis and drug delivery. Considering the importance of understanding the transport and deposition of microparticles, people have conducted extensive research [5,6,7,8,9,10,11]. However, most of the research objectives are still in the upper respiratory system, and there are relatively few studies on the alveolar region. Due to the difficulty of establishing a real physical model for particle transport and deposition in alveoli, numerous studies focused on the improvement of numerical method [12,13,14,15,16,17,18,19,20].

Several experimental studies using enlarged alveolar models have been proposed to study the alveolar flow and particle transport in the alveolar region [21,22,23,24,25,26]. Ma et al. [24] and Berg et al. [25] used the scaled-up model to track the particles in the ducts. Chhabra and Prasad [26,27,28] in their studies focused on the deposition regions of the particles. These researchers have made great contributions to display the fates of particles in the alveoli. However, it is hard to simultaneously match the dynamic similarity between alveolar flow and particle motion in enlarged alveolar models [29]. Therefore, there are still broad prospects for improvement in experimental methods to reveal the fluid and microparticle behaviors in alveoli under the condition of actual size.

Based on the microfluidic technology, human alveoli-on-a-chip was built to study the fluid flow and particle transport and deposition. Sznitman’s group [30,31,32,33] developed a 5-generation alveolar chip in real size. By controlling the pressure in the surrounding chambers, the periodical expansion and contraction of the channel wall was achieved. Two kinds of flow patterns in the alveoli were measured using Micro-Particle Image Velocimetry (Micro-PIV) technique [30]. Fishler et al. [31] tracked the trajectories of the smoke particles in a 5-generation chip. However, the maximum tracking time is one period. This study mainly revealed the characteristics of particles penetrating into the alveoli. The subsequent work by Fishler et al. [33] revealed the trajectories of particles in ducts and alveoli. Their work can track the particles for several periods in the alveoli. However, their results are in agreement with the results of massless tracers. The detailed particle motions in an alveolus under various forces were not presented. For the real particles, the gravity also has large impacts on the particle transport besides the drag force and diffusion, because larger particles can also deposit in the alveoli due to gravitational sedimentation [34,35]. According to the previous studies [1,26,36,37], the directions of the alveolar openings vary across the whole lungs. On the other hand, according to Lv et al. [38] and Dong et al. [39], compared with multi-generation alveolar chip, the alveolar chip with single alveolus has the advantage in closely matching the flow parameters with the real ones. Therefore, the effects of forces, such as the gravity force, drag force as well as the direction of the gravity force, on the particle transport in an alveolus should be evaluated.

In the present study, single alveolus on a chip was designed to obtain more detailed particle trajectories and deposition in alveoli. The respiratory behaviors were precisely mimicked by independently and simultaneously controlling the fluid flow and expansion of the alveolar wall through two separate channels and syringe pumps. More details about the alveolar chip design and control methods could be found in our earlier work [38]. We apply a glycerol/water solution mixed with solid particles to acquire multi-period particle trajectories of higher image resolution. Besides the dynamic similarities of fluid flow, the similarities of particle motion were also matched with the particles in the human lung by matching dimensionless numbers. Our design, that is, the normal direction of the alveolar chip is perpendicular to the direction of gravity, can overcome the deviation of particles from the focal plane due to the influence of gravity. This design ensures that we do not lose particles in the process of tracking them. The effects of chaotic flow in alveoli, gravity, gravity directions, and fluid drag force were evaluated. Through changing the orientation of the alveolar opening in the alveolar chip, we can study the different effects of gravity and drag force, which is of great significance to understand the movement of particles in the alveoli with different opening orientation in the human body. Our experimental observations could provide new insight into particle trajectories and deposition in alveoli.

## 2. Materials and Methods

### 2.1. Experimental System

**Figure 1:**
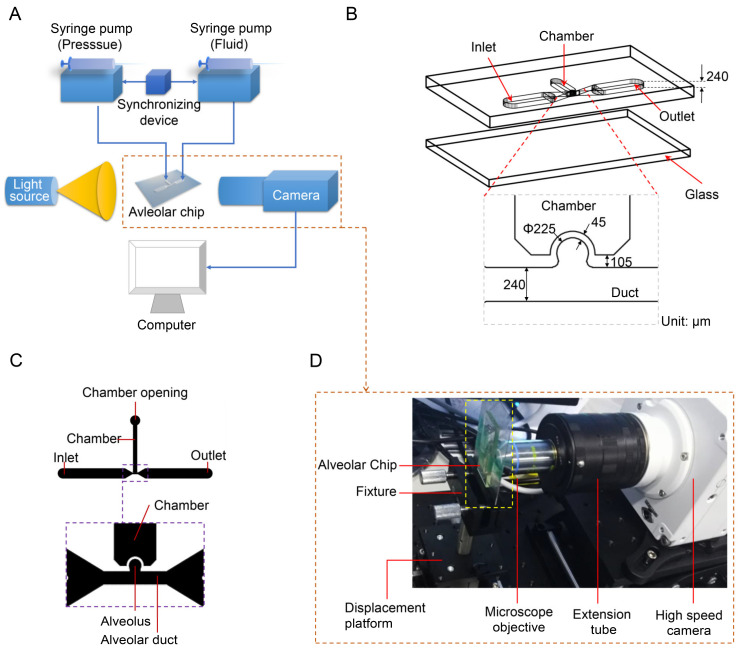
Model of the alveolus chip and experimental system. (**A**) Schematic diagram of experimental system. (**B**) Schematic diagram of the alveolar chip. (**C**) CAD drawing of the alveolar chip. (**D**) Photo of optical observation system. Figure (**C**) is adapted from the work of Lv et al. [38]. [Original citation]—Reproduced by permission of The Royal Society of Chemistry.

The experimental system is mainly composed of three parts: an alveolar chip, a flow control system, and an optical system. The schematic diagrams of the experimental system, the alveolar chip and the computer-aided design (CAD) drawing of the chip are shown in Figure 1A–C, respectively. As shown in Figure 1B, the quasi-three-dimensional alveolar model based on microfluidic chip is adopted as a substitute for a three-dimensional spherical alveolar model to study the transport behaviors of microparticles in the alveolus. The alveolar chip is designed according to the anatomical structure and the dimensions of the alveolus in the last generation (i.e., the 23rd generation of 0th~23rd generations) in human body. The alveolar chip is fabricated using the soft lithography technology and cast in a mixture of polydimethylsiloxane (PDMS)/curing agent with 10:1 (*w/w*). The microfluidic device is similar to our recent published work [38] and the fabrication details can be found from it. The geometrical parameters for the alveolar duct, alveolus and the morphometric arrangement of the airways come from the previous studies [36,37,40,41]. The chip mainly consists of a partial cylindrical alveolus, an alveolar duct with a square cross-section and a pressure control chamber with a rectangular cross-section, as shown in Figure 1B,C. The alveolar diameter (*D*_a_), the ductal width (*W*) and the ductal height (*H*) of the alveolar chip are 225 μm, 240 μm and 240 μm, respectively. The reliability of using this quasi-three-dimensional model as an alternative to the three-dimensional spherical model for flow field experiments has been verified by Lv et al. [38]. Note that the single alveolar chip is used without considering the interaction between different alveoli and the difference among so many alveoli in the same generation. The elasticity of PDMS material can realize the deformation of alveolar wall, and the transparency can meet the requirements of flow visualization. Although the physical parameters of PDMS, such as elastic modulus, are different from those of the alveolar membrane, it can still meet the experimental requirements as long as we make the expansion coefficient of the alveolus in the chip close to that of the real alveoli [30].

The control system is used to precisely control the fluid flow and the deformation of alveolar walls, which can mimic the respiratory behaviors of human alveoli and achieve dynamic similarity. The control system has two syringe pumps TYD01 (Lead Fluid Technology Co., Ltd., Baoding, China), a plastic syringe, a microinjector and a synchronizing device. The plastic syringe is mounted on one syringe pump and linked to the pressure control chamber of the alveolar chip by a stainless-steel needle (inner diameter 0.51 mm, outer diameter 0.8 mm) and a PTFE (polytetrafluoroethylene) catheter. The pressure in the chamber varies periodically, which is controlled by the syringe pump, causing the rhythmic expansion and contraction of the alveolar wall. The other syringe pump is applied to control the microinjector to inject and extract the mixed solution with particles into and out of the alveolar duct through the inlet on the chip. The two syringe pumps are connected by a synchronizing device to coordinate and cooperate to realize the periodic breathing movement of the alveolus and dynamic similarity of fluid flow in the alveoli.

The optical system as shown in Figure 1D is used to visualize the trajectories of particles. It mainly consists of an Olympus microscope objective LCACHN 40XPH, UIS2 (Olympus, Tokyo, Japan), a Photometrics high-speed camera Phantom VEO 710L (Vision Research Inc., Wayne, NJ, USA), as well as some necessary optical table equipment. The objective lens used in the experiment has a numerical aperture of 0.55 and a working distance of 2.2 mm. The photos are taken by high-speed camera with a frame rate of 24 fps (i.e., the time resolution is 0.04 s). The collected photos are post-processed using ImageJ 1.52a (National Institutes of Health, Bethesda, MD, USA) and Image-Pro Plus 6.0 (Media Cybernetics, Inc., Rockville, MD, USA) is used to obtain trajectory coordinates of particles at different time.

**Figure 2:**
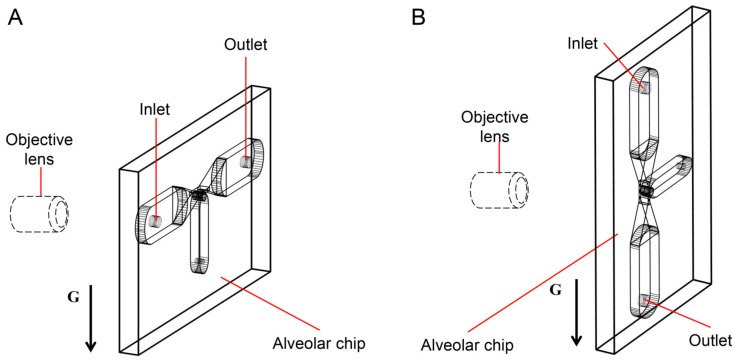
The gravity directions. (**A**) Perpendicular to the axis of the alveolar duct. (**B**) Parallel to the axis of the alveolar duct.

Assuming that the alveolar chip is placed horizontally on the microscope platform, the objective lens of the inverted microscope coincides with the gravity direction. Therefore, we can only observe the behaviors of particles on one horizontal plane in the image, and cannot study the influence of gravity on the particles. To solve this problem, the chip (the part enclosed by a yellow rectangle in Figure 1D) is placed parallel to the direction of gravity in this experiment. The objective lens is perpendicular to the plane of the chip and is connected to the high-speed camera through a long barrel. The high-speed camera is connected to a computer. As Figure 2A,B shows, the alveolar chip is placed in two directions to study the effect of gravity directions on the particle transport in the alveolus. In this way, we can compare the differences of particle behaviors when the direction of the gravity is parallel or perpendicular to ductal fluid flow.

### 2.2. Modeling the Fluid Flow in Alveoli

Instead of using smoke to study the movement and deposition of fine particles [31], we aim to apply a glycerol/water solution mixed with particles to acquire more flexibility for flow control and higher image resolution for optical visualization. However, considering the different properties between the air and the glycerol/water solution, it is necessary to achieve dynamic similarity between the glycerol/water solution flow and the air flow to model the real alveolar flow and particle transport.

The dynamic similarity is achieved by setting the same Reynolds number (*Re*, $Re=\frac{uD_{d}}{v}$, *u* is the flow velocity in the duct; *D*_d_ is the characteristic length of the duct, i.e., the ductal diameter in human lung, which equals to the width (*W*) of the alveolar duct in the chip; and *ν* is the kinematic viscosity of the flow) and Womersley number (*Wo*, $Wo\;=\;D_{d}\sqrt{\frac{2\pi }{Tv}}$, *T* refers to the breathing period) between the glycerol/water flow in the alveolar chip model and the air flow in the real alveoli [30,31,42]. When the fluid flow rate and breathing period are matched with those of real situation, the key is to match the kinematic viscosity of the selected liquid with that of air. Similar to the experiments of Fishler et al. [30] and Lv et al. [38], we prepared a mixture solution of a 36:64 (*v/v*) glycerol/water, whose kinematic viscosity (*ν*_mix,24 °C_ = 1.65 × 10^−5^ m^2^/s) is matched closely with that of the 24 °C air (*ν*_air,24 °C_ = 1.67 × 10^−5^ m^2^/s). We selected the fluid flow properties in alveoli at the 23rd and 21st generations with the two kinds of representative flow patterns to study the particle transport behaviors. The respiratory cycle is 4 s, which approximately equals to the quiet breathing cycle of human lung. The ductal flow rate in alveolar duct at the 21st and 23rd generations are 0.13 μL/s (Re = 0.032) and 0.02 μL/s (Re = 0.0053), respectively, at the peak respiration. The corresponding alveolar to ductal flow rates are 0.63 at the 21st generation and 4.25 at the 23rd generation, respectively.

### 2.3. Modeling the Particle Transport in Alveoli

Previous studies [43] showed that particles smaller than 0.5 μm in diameter are mainly deposited by diffusion, while particles larger than 0.5 μm in diameter are mainly deposited by external forces. In this paper, we focus on the transport behaviors of particles with an average diameter of 0.5 μm which is the critical diameter for particles in the human alveoli.

Fine particles are subjected to multiple forces in a fluid flow. When we replace the air with the glycerol/water solution, we also need to verify the dynamic similarity of particle transport in it apart from fluid flow dynamic similarity mentioned above. In order to simplify the experimental conditions, it is assumed that the particle size is uniform, and the average diameter is 0.5 μm. The fluid flow rates on both sides of the particles are the same, and the deformation and rotation of the particles themselves are not considered here. Therefore, Maguns force, buoyancy, additional mass force and Saffman force are ignored. The governing equation for fine particles in a fluid flow can be simplified to [41],

(1) $$m\frac{du_{p}}{d_{t}}=F_{D}+F_{B}+F_{g}$$

where ***u***_p_ is the particle velocity, $F_{D}$ is the drag force that is generated by the viscous behaviors between the particles and the fluid, $F_{g}$ is the gravity, and $F_{B}$ is the Brownian force. Considering that the Brownian behaviors of the tiny particles only need to be considered when the particle size is close to the air mean free path (about 0.07 μm), the Brownian force is ignored for particles with size of 0.5 μm. Therefore, the governing equation becomes:

(2) $$m\frac{du_{p}}{d_{t}}=F_{D}+F_{g}.$$

Three dimensionless numbers pertaining to the particle dynamics are evaluated and their values in liquid flow must be equal to the values in the air flow in order to achieve the particle dynamic similarity between the experiment and the real case. The dimensionless numbers are Stokes number (*Stk*, characterizing the importance of fine particle inertia in the fluid flow and being related to the drag force), Gravity number [41,44] (*H*, characterizing the importance of gravity sedimentation) and particle Peclet number (*Pe*_p_, characterizing the ratio of convection transport rate to diffusion transport rate of the particles):

(3) $$Stk=\frac{\omega \rho_{p}d_{p}^{2}C_{c}}{18\mu }\;$$

(4) $$H=\frac{g\rho_{p}d_{p}^{2}C_{c}}{9\mu D_{d}\omega }$$

(5) $$Pe_{p}=\frac{D_{d}^{2}\omega }{D_{mol}}=\frac{3\pi D_{d}^{2}\mu d_{p}}{k_{B}T_{em}C_{c}}$$

where *ω* = 2π/*T* is the breathing frequency, *ρ*_p_ is the density of the particles, *g* is the gravitational acceleration, *d*_p_ is the particle diameter, *μ* is the dynamic viscosity of fluid, *D*_mol_ is the diffusion constant of spherical particles, *k*_B_ = 1.38 × 10^−23^ J/K is Boltzmann’s constant, *T*_em_ is the temperature in Kelvin, *C*_c_ is the Cunningham slip correction factor [45] and approximate formula for *C*_c_ when *d*_p_ > 0.1 μm is *C*_c_ = 1 + 2.52 *λ*/*d*_p_, *λ* is the mean free path of molecules in the fluid. For air, the mean free path at room temperature and 1 atm pressure is 0.067 μm. The molecules in liquid are very close together and the mean free path is smaller than the average inter-molecular spacing. Therefore, we ignore the mean free path for the glycerol/water solution for simplification. According to Equations (3)–(5), we find that the fluid dynamic viscosity is included in these equations. Note that the dynamic viscosities of the air and the glycerol/water solution are *μ*_air,24 °C_ = 1.79 × 10^−5^ Pa∙s and the *μ*_mix,24 °C_ = 1.77 × 10^−2^ Pa∙s, respectively. The dynamic viscosity of the glycerol/water solution is about 1000 times that of the air. The three equation indicate that the particle dimensionless numbers cannot be matched simultaneously. The *Pe*_p_ values of particle in air flow and glycerol/water solution are 1.4 × 10^3^ and 1.8 × 10^7^, respectively. We make a compromise solution match only *Stk* and *H* which are closely related to drag force and gravity, respectively. In order to match both *H* and *Stk* numbers of particles in glycerol/water solution with those in the air, our solution is to increase both the density and diameter of particles in the liquid by 10 times.

We chose silver particles with a diameter of 5 μm and density of 10.5 g·cm^−3^ as a substitute in the glycerol/water solution in the alveolar chip to represent the typical particles with a diameter of 0.5 μm and a density of 1 g/cm^3^ [41] in the air in the real acinus. The values of the dimensionless numbers of the particles are listed in Table 1. *Stk* and *H* of the 0.5 μm particles in air in the real acinus are about 1.25 times that of the 5 μm particles in glycerol/water solution in the alveolar chip. The silver powder is mixed evenly with the water and glycerin solution at a rate of 0.6 g/L, and the surfactant Triton X-100 (Beijing Solarbio Corporation, Beijing, China) solution is diluted in a volume ratio of 500:1 to prevent aggregation of the silver particles.

## 3. Results and Discussion

### 3.1. Mapping Particle Trajectories

**Figure 3:**
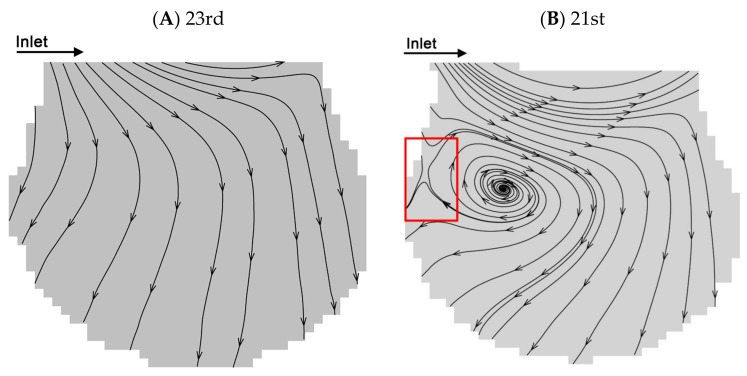
Flow patterns at the 21st and 23rd generations. (**A**) Radial flow pattern at the peak inspiration at the 23rd generation. (**B**) Recirculation flow pattern at the peak inspiration at the 21st generation. Figures (**A**–**B**) are adapted from the work of Lv et al. [38]. [Original citation]—Reproduced by permission of The Royal Society of Chemistry.

Two typical flow patterns which are reproduced from the work of Lv et al. [38] are shown in Figure 3A,B. We use the same alveolar chip and flow parameters. According to previous studies [22,30,38], there are two typical flows in the alveoli, i.e., radial flow and vortex flow. The fluid flow properties in alveoli at the 23rd and 21st generations with the two kinds of representative flow patterns are selected to study the particle transport behaviors. According to the streamlines, the fluid elements flow toward the direction perpendicular to the ductal direction in the radial flow pattern at the 23rd generation. The recirculation flow pattern at the 21st generation contains the radial flow and the vortex flow. The flow elements also flow toward the direction parallel to the ductal direction. Furthermore, the recirculation flow pattern contains a critical region (i.e., the part enclosed by a red rectangle in Figure 3B) with a saddle point.

**Figure 4:**
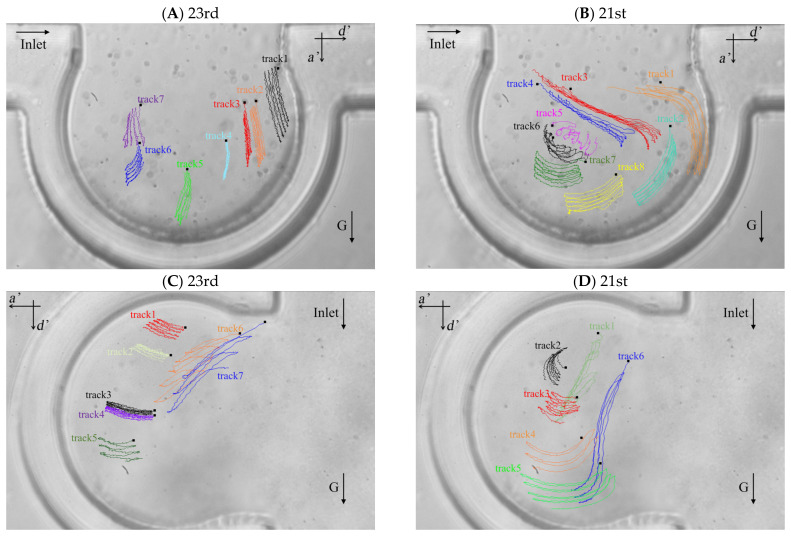
Particle trajectories in the alveolar chip. (**A**) Particle trajectories in the alveolus of the 23rd generation with gravity direction perpendicular to the ductal flow. (**B**) Particle trajectories in the alveolus of the 21st generation with gravity direction perpendicular to the ductal flow. (**C**) Particle trajectories in the alveolus of the 23rd generation with gravity direction parallel to the ductal flow. (**D**) Particle trajectories in the alveolus of the 21st generation with gravity direction parallel to the ductal flow.

The particle trajectories of five respiratory cycles in the alveolus of the 23rd and 21st generations based on the two typical flow fields are shown in Figure 4A–D. We defined two directions for the particle motion, the one of which is perpendicular to the direction of the ductal flow (i.e., the direction of a’ axis in Figure 4) and the other one is parallel to that (i.e., the direction of d’ axis in Figure 4). The positive displacements are the positive directions of a’ axis and d’ axis. Lines with different colors represent the trajectories of different particles and are labeled track1, track2, track3, etc. The initial location of each trajectory is shown as a small black square. Figure 4A,B show the trajectories of particles in the alveolus of the 23rd and the 21st generations, respectively, when the direction of gravity is perpendicular to the direction of ductal flow. Figure 4C,D show the trajectories of particles in the alveolus at the 23rd and 21st generations, respectively, when the direction of gravity is parallel to the direction of the ductal flow. In this experiment, the Stokes number (*Stk*) of silver particles in the fluid flow is in the order of 10^−6^ which is much smaller than 1, indicating that the particles should follow the streamlines exactly. The results from the work of Tsuda et al. [42] showed that the trajectories of massless particles show reversibility except for the trajectories in a “critical region” (in the red rectangle in Figure 3B) near the saddle point in the recirculation flow pattern. However, our results show that the trajectories throughout the alveolus show irreversibility. The particles not only move along the streamline (Figure 3), but also settle along the direction of gravity. The offset of the trajectory of the particle between different periods is caused by the gravity. The drag force is the main mechanics for particle motion in several periods, which is supported by comparing the length of the particle trajectories with offset of the trajectories, such as track3, track4, track7 and track8 in Figure 4B, track1, track2, track3, track4 and track5 in Figure 4C and track3, track4 and track5 in Figure 4D. The particles move further along the streamline than in the direction of gravity in a few periods. The particle trajectories at the 21st generation are longer than that at the 23rd generation, indicating that the velocity magnitude in the alveolus of 21st generation is relatively larger. Although the effect of gravity is relatively less than the effect of drag force, the gravity has a decisive contribution to the deposition of particles.

### 3.2. Statistical Average Particle Displacement

**Figure 5:**
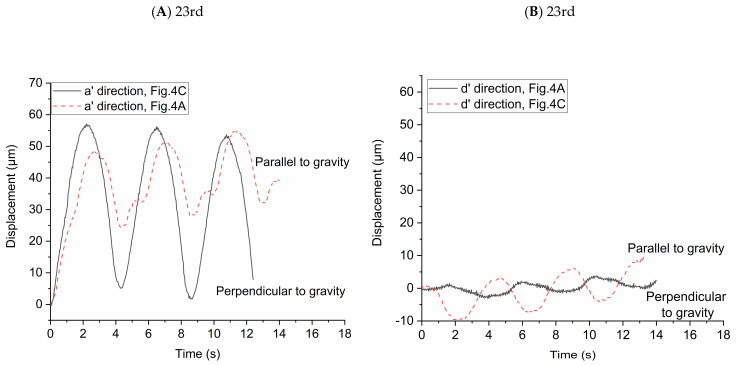

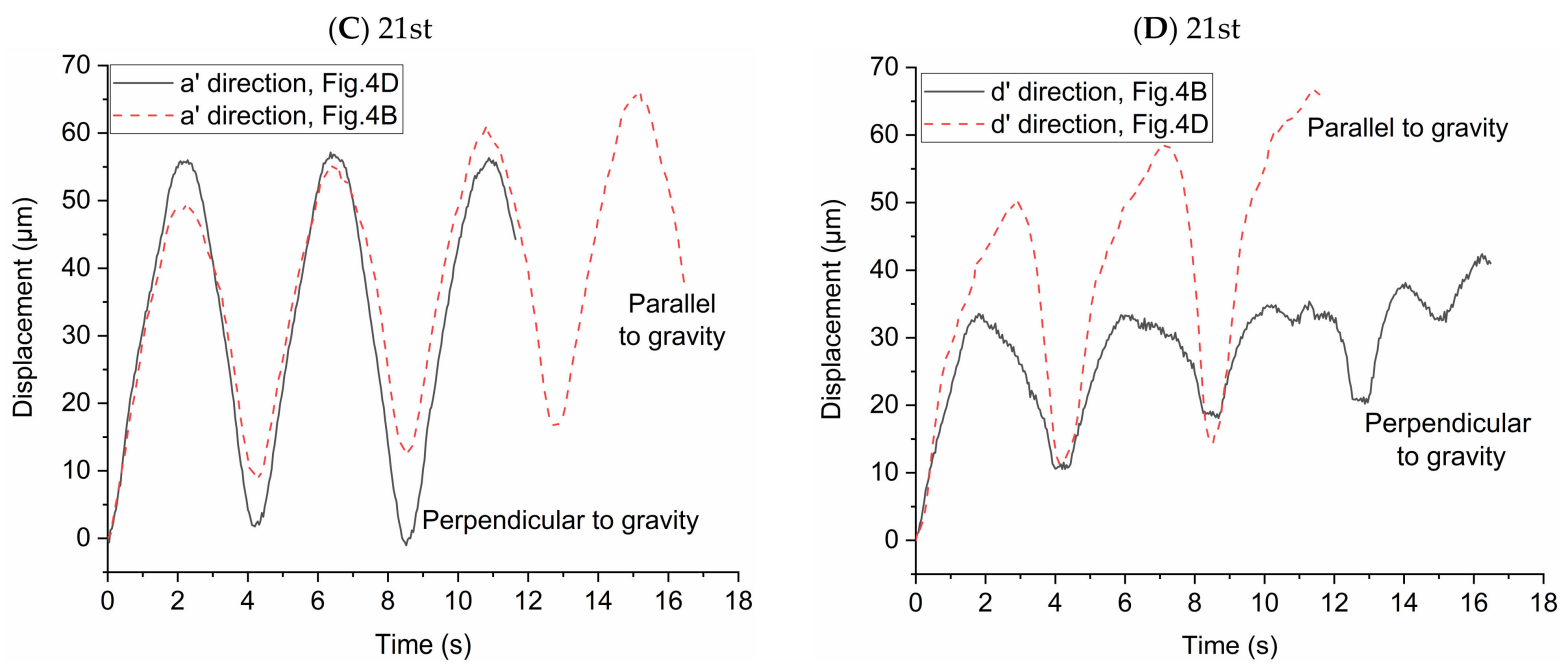
Statistical average displacement of particles in two directions under zero and normal gravity. (**A**) Displacement of particles in the a’ direction at the 23rd generation. (**B**) Displacement of particles in the d’ direction at the 23rd generation. (**C**) Displacement of particles in the a’ direction at the 21st generation. (**D**) Displacement of particles in the d’ direction at the 21st generation.

In order to study the influence of gravity on particle transport behaviors, the particle displacements relative to the initial location of each particle in two directions (i.e., the direction of a’ and d’ axes) were investigated. The high-speed camera was used to take pictures every 0.04 s and we used the Image-Pro Plus 6.0 (Media Cybernetics, Inc., Rockville, MD, USA) to get the locations of the particles in the two directions in each picture. We averaged the displacements of all the particles in the pictures to observe the overall trend of particle displacement in the two directions. In Figure 4A,B, the effect of gravity on the particle displacement is in the direction of a’ axis. In Figure 4C,D, the effect of gravity on the particle displacement is in the direction of d’ axis. The statistical average displacements of particles in the two directions are shown in Figure 5. Lines in the figures indicate the direction of particle displacement is perpendicular to the gravity and dashed lines indicate the direction of particle displacement is parallel to the gravity. When the direction of particle displacement is perpendicular to the gravity, the gravity has no effect (i.e., zero gravity) on the particle motion in this direction. However, the gravity has the most effect (i.e., normal gravity) on the particle motion in the direction that particle displacement is parallel to the gravity. Therefore, the particle motion under zero gravity and normal gravity can be quantified based on this method.

Figure 5A shows the statistical average displacements of particles in a’ axis direction under zero gravity and normal gravity in Figure 4A,C, respectively. When the statistical average particle displacement in a’ axis direction is perpendicular to the gravity (in Figure 4C), the particles transport is only due to the drag force in this direction. The statistical average displacement of particles is like a sinusoidal curve. The vertexes and valleys of the curve are the statistical average displacement of particles at the end of inspiration and the end of expiration, respectively. When the statistical average particle displacement in a’ axis direction is parallel to the gravity (in Figure 4A), both drag force and gravity affect the particle transport. The statistical average displacement is no longer a regular sine sinusoidal curve. The values of the vertexes and valleys of the curve increase with the increase of the number of respiratory cycles, indicating the particles move towards the alveolar wall in the a’ axis direction. Figure 5B shows the statistical average displacement in the d’ axis direction under zero gravity and normal gravity in Figure 4A,C, respectively. The two curves change in opposite directions. The displacements are much smaller because the fluid mainly flows along the a’ axis direction due to the radial flow pattern. When the particle displacement in d’ axis direction is parallel to the gravity (in Figure 4C), the values of the vertexes and valleys of this curve increase obviously as the number of respiratory cycles increases. Furthermore, this figure shows that the statistical average displacements represented by the two curves are not always large than zero in a few cycles, indicating the motion of each particle is not unidirectional in this direction relative to the initial position in the radial flow pattern. Figure 5C shows the same results as Figure 5A, even if they are in different flow field in the alveolus. In Figure 5D, the statistical average particle displacement in d’ axis direction is still obvious due to the vortex flow field, which is different from the result of Figure 5B. In Figure 5D, the statistical average particle displacement in the d’ direction parallel to the gravity (in Figure 4D) is almost the similar to that in the a’ direction parallel to the gravity in Figure 5C, which shows that the effect of the drag force and gravity have no large bias in the two direction. However, the statistical average particle displacement in the d’ direction perpendicular to the gravity (in Figure 4B) is much smaller. It is because some particles (for example, track1 in Figure 4B) move in the negative direction of the d’ axis, while other particles move (for example, track2 in Figure 4B) in the positive direction of the d’ axis. In general, the drag force contributes to the large reversible transport of particles, while the gravity contributes to the particle deposition.

### 3.3. Displacements of Individual Particles

**Figure 6:**
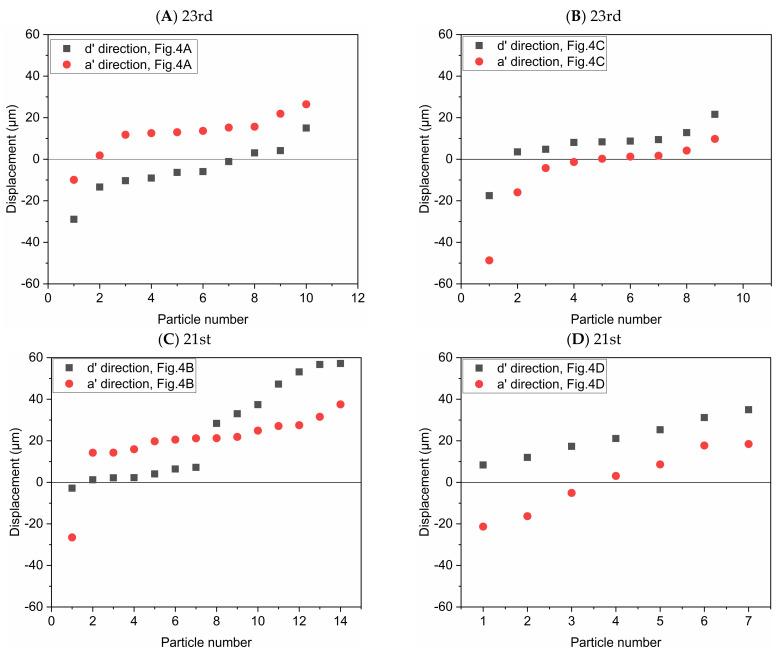
Displacement of the particles after 5 periods in the direction parallel or perpendicular to gravity, respectively. The alveolus arrangement is the same with Figure 4, which respectively corresponds to (**A**) the 23rd generation in Figure 4A; (**B**) the 23rd generation in Figure 4C; (**C**) the 21st generation in Figure 4B; (**D**) the 21st generation in Figure 4D.

In order to study the possibility of particle deposition in the alveoli or escaping from it, the displacements of individual particles after multiple periods are shown in Figure 6. The alveolus arrangement is the same with that in Figure 4. We arranged the data in order of the displacement magnitude. Horizontal line with displacement equal to 0 represents their initial positions. These circles and squares represent the displacement relative to the initial positions. Deviation from the initial position means that particles may deposit in the alveolus or escape from it according to the displacement direction, i.e., positive or negative displacement. As presented in Figure 4, positive displacement in a’ direction usually means deposition, while negative one indicates escaping from the alveolus. Figure 6A shows that the particles tend to move in the positive direction of a’ and in the negative direction of d’, which indicates that most of the particles cannot escape from the alveoli with the increase of the number of cycles. Figure 6B shows that the particles tend to move in the positive direction of d’ and in the negative direction of a’. Although gravity would help the deposition of particles, many particles still escape from the alveoli due to the effect of drag force. Figure 6C shows that the particles tend to move in the positive direction of a’ and d’. The vast majority of the particles will be trapped in the alveolus. The particles are deposited towards the alveolar wall in the positive direction of d’, which is opposite to the result shown in Figure 6A. The results shown in Figure 6D are basically similar to those in Figure 6B. Therefore, the direction of gravity as well as the different flow patterns between 21st and 23rd generation have a great influence on the particle deposition and escaping from the alveoli.

## 4. Conclusions

Applying an alveolar chip to study the particle transport in an alveolus was provided in this study. The alveolar chip could mimic the expansion and contraction of human alveoli of different generations and the dynamic similarity between the alveolar chip and human acinus was achieved. An innovative experimental method of applying silver particles mixed in glycerin solution to replace fine particles suspended in the air was proposed, which gave us more flexibility for flow control and better image resolution for optical visualization. The normal direction of the alveolar chip is perpendicular to the direction of the gravity, which can ensure that we track the particles for a long time without losing their images. The experimental platform could change the orientation of the chip to study the effect of gravity on particle transport. Through tracing the trajectories of particles in five breathing cycles, the influence of drag force and gravity on particle transport was thoroughly investigated for different orientations of the chip and generations. The experimental results show that the drag force is the main mechanics for particle reversible motion. Although the effect of gravity on particle motion is relatively less important than the effect of drag force, the gravity has a decisive contribution to the deposition of particles. The possibility of particles leaving the alveoli is related to the direction of gravity. Moreover, drag force can help particles escape. We found that the changes in the flow pattern will also change the direction of particle deposition. This study provides valuable data for the studies of particle transport in alveoli, and we expect that the proposed experimental methods will become a useful tool for follow-up studies on drug delivery and screening.

## Figures and Tables

**Table 1:** Comparison of dimensionless numbers for particles.

*i*th Gen.	Alveolar Chip with 5 μm Particles		Real Acinus with 0.5 μm Particles	
	*Stk* ^1^	*H* ^1^	*Stk* ^2^	*H* ^2^
21	1.29 × 10^−6^	0.043	1.63 × 10^−6^	0.046
23	1.29 × 10^−6^	0.043	1.63 × 10^−6^	0.054

^1^ The dimensionless number of particles in the alveolar chip; the alveolar duct of the alveolar chip at the 21st and 23rd generations have the same square cross section with sides that are 240 μm in length. ^2^ The dimensionless number of particles in the real acinus; the diameters of alveolar duct in the real acinus at the 21st and 23rd generations are 280 μm and 240 μm, respectively.

## Data Availability

Not applicable.
